# Effective Use of Sugarcane-Bagasse-Derived KOH-Activated Biochar for Remediating Norfloxacin-Contaminated Water

**DOI:** 10.3390/toxics11110908

**Published:** 2023-11-06

**Authors:** Yan Zhou, Yongtao Lan, Michael Douglas Short, Juanjuan Shi, Qiugui Zhang, Junhao Xu, Gujie Qian

**Affiliations:** 1 School of Ecology and Resource Engineering, Wuyi University, Wuyishan 354300, China; 12138smg@gmail.com (Y.L.); xjh218573@163.com (J.X.); 2 Arris Pty. Ltd., Urrbrae, Adelaide, SA 5064, Australia; 3 Future Industries Institute, Science, Technology, Engineering and Mathematics (STEM), University of South Australia, Mawson Lakes, Adelaide, SA 5095, Australia; 4 Zhejiang Academy of Agricultural Sciences, Hangzhou 310021, China; sjj1998wyu@163.com; 5 Fujian Hengxiang Co., Ltd., Songxi 353500, China; zqg299625@163.com; 6 College of Science and Engineering, Flinders University, Bedford Park, Adelaide, SA 5042, Australia

**Keywords:** Chinese sugarcane bagasse, KOH activated, biochar, adsorption, norfloxacin removal

## Abstract

Bagasse-derived biochar (SCB_750_) was prepared at 750 °C using Chinese sugarcane bagasse as a carbon source and then modified with KOH for the removal of the antibiotic norfloxacin (NOR) from aqueous solutions. 3K-SCB_750_, prepared using a solid-to-liquid mass ratio of bagasse:KOH = 1:3, was found to have the best adsorption performance for NOR. Under the conditions of pH 5, 25 °C, 2.4 g L^−1^ adsorbent, and 300 mg L^−1^ NOR, its adsorption of NOR reached equilibrium (97.5% removal) after 60 min. The adsorption behaviours were in line with the quasi-second-order kinetic and Langmuir isotherm models, respectively. The maximum theoretical adsorption capacity reached up to 157.4 mg·g^−1^ at 40 °C. The thermodynamic parameters showed that the adsorption of NOR onto 3K-SCB_750_ was a spontaneous, endothermic, and physical process. In addition, Brunauer−Emmett−Teller analysis (BET), scanning electron microscopy (SEM), transmission electron microscopy (TEM), X-ray diffraction (XRD), and Raman spectroscopy were conducted to investigate the structural and adsorption properties of 3K-SCB_750_. Fourier transform infrared spectroscopy (FTIR) was also applied to understand the mechanism of adsorption of NOR onto 3K-SCB_750_. All of the results indicated that 3K-SCB_750_ had a large specific surface area of 1038.8 m^2^·g^−1^, an average pore size of 1.9 nm, and hierarchical structures with random pores and cracks for efficient removal of NOR. NOR adsorption mechanisms on 3K-SCB_750_ were related to the pore-filling effect and electrostatic attraction. Therefore, 3K-SCB_750_ biochar may be used as a promising adsorbent of antibiotics in wastewaters.

## 1. Introduction

Although antibiotics afford substantial benefits to human and animal health, global overuse of antibiotics both clinically and in animal production have created problems regarding pollution of the receiving environments [1] and the promotion of antimicrobial resistance, which is now a UN-designated global health crisis, causing millions of deaths per year and costing the global economy over USD 3 trillion annually [2]. Consequently, intensive efforts are underway globally to better manage the use of antibiotics and reduce the amounts discharged to receiving environments from various waste streams [3,4]. Norfloxacin (NOR), a fluoroquinolone antibiotic, is extensively used clinically and in livestock breeding to inhibit Gram-positive and Gram-negative bacteria [5]. Recently, NOR has become one of the most widely used antibiotics, due to the rapid development of quinolone antibiotics [6]. The absorption efficiency of these antibiotics by humans and animals is low, meaning that 60–70% of the applied active compound is discharged to receiving environments through feces and urine [7]. Because of the growing concern of environmental pollution with residual NOR and implications for the emerging threat of antimicrobial resistance [8], there is a need to develop new and effective techniques for the removal of NOR contaminants from waste streams.

Adsorption is one of the most efficient strategies for the removal of antibiotics from aqueous media. The commercially available activated carbons are expensive and therefore not suitable for application on a large scale. To reduce the cost of biochar (porous carbons) adsorbents and enhance their sorption performances, much effort has been made regarding the development of cost-effective porous carbon materials derived from renewable sources. Various biomass waste materials have been widely utilized as precursors of biochar, such as bamboo sawdust, cassava waste, coffee grounds, and reed [9,10,11,12]. Recently, biochar has been extensively studied and applied in the fields of pollutant treatment and water purification, owing to its porous structures, large specific surface areas, large pore volumes, and abundant functional groups [13].

The global production of sugarcane totaled 1.9 billion tones in 2019, being the world’s third most-produced commodity [14]. Considering that every ton of sugarcane generates around 270 kg of sugarcane bagasse by-product, the 2019 worldwide harvest produced approx. 526 million tons of sugarcane bagasse, which is currently an under-utilized by-product stream requiring the development of new and valuable applications [14]. Taking 2020 as an example, China planted about 1.4 million hectares of sugarcane with an annual production of about 110 million tons, resulting in some 50 million tons of sugarcane by-products [15]. The conversion of such large amounts of sugarcane waste to carbon-rich biochar may open a new avenue for the reutilization of sugarcane waste products for advanced treatment applications in polluted wastewaters. However, pristine biochar materials generally have relatively small specific surface areas and poor adsorption performances. Hence, biochar is often subjected to physical and/or chemical activation to improve the adsorption properties. Chemical activation methods generally require lower energy consumption and less time compared with physical activation methods [16], and so offer a more sustainable pre-treatment approach. Among the chemical activators, KOH has been most applied due to its effective intercalative property and excellent pore-forming efficiency and quality [17].

Several studies have reported the utilization of biochar for the removal of NOR [9,10,11,12]. However, to date, there has been little or no information regarding the application of sugarcane-waste-based biochar for NOR removal. Therefore, the aim of this study was to use the readily available sugarcane waste as a carbon source to prepare porous biochar materials via a simple and cost-effective route, combining carbonization with KOH activation in one step. The specific objectives were to: (1) investigate the physio-chemical properties of the biochar; (2) investigate the adsorption performance of NOR under various experimental conditions (contact time, solution pH, initial concentration, and temperature conditions); and (3) determine the possible mechanisms of the formulated biochar for NOR removal. This work showcases a novel utilization of sugarcane waste material and provides a scientific basis for its application in NOR removal from antibiotic wastewater.

## 2. Materials and Methods

### 2.1. One-Step Synthesis of KOH-Modified Biochar

**Figure 1:**
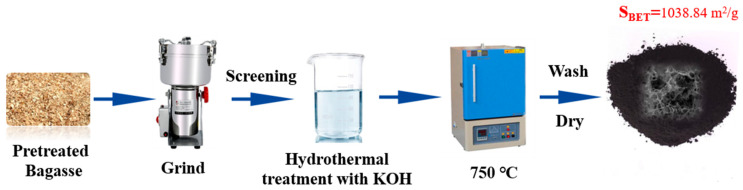
Schematic illustration of the synthetic process of 3K-SCB_750_.

Locally available sugarcane bagasse (Minsheng Health Technology Company, Songxi, Fujian Province, China) was used in this study. Figure 1 illustrates the synthetic process for modified biochar. Here, 40.0 g of bagasse was first dispersed in 200 mL of deionized water with magnetic stirring at 600 rpm for 3 h to remove dust and impurities, followed by oven drying at 70 °C. Then, the pretreated bagasse was ground (BJ-800A, Duojie Equipment Co., Ltd., Huzhou, China) and dry-screened to <75 µm. The ground powders were subsequently impregnated into a KOH solution (solid-to-liquid mass ratio of bagasse-to-KOH = 1:1, 1:2, or 1:3, hereafter referred to as 1K-SCB_750_, 2K-SCB_750_, and 3K-SCB_750_) at room temperature, left for 24 h, and then heated in a furnace at 750 °C for 60 min at a heating rate of 5 °C min^−1^. Finally, the furnace was turned off and cooled down to room temperature. The resulting samples were rinsed several times using hot deionized water (90–100 °C) until the pH of the wash water was near-neutral, followed by drying at 105 °C for about 12 h. Note: we only used three bagasse-to-KOH ratios (1:1 to 1:3) for preparing the KOH-modified biochar materials, as using more KOH (e.g., 1:10 bagasse-to-KOH ratio) would potentially increase the manufacturing cost. Based on this reason, we did not attempt to investigate the optimal ratio for synthesizing the KOH-modified biochar.

### 2.2. Batch Adsorption Experiments

To compare the adsorption capacity of the unmodified (B_750_) and modified biochars (1K-SCB_750_, 3K-SCB_750_, and 3K-SCB_750_) and select the best modified biochar material for the following work, preliminary adsorption experiments were carried out using 50 mg of each sample placed in a 50 mL plastic tube containing 25 mL NOR solutions (initial concentration: 300 mg·L^−1^) without pH adjustment and shaken at 300 rpm at 30 °C for 30 min.

Following these preliminary adsorption experiments, the best KOH-modified (i.e., 3K-SCB_750_; see Results in Section 3.2.1) and the unmodified biochar materials were chosen for detailed investigation and comparison of their capabilities of NOR adsorption using batch experiments conducted under the conditions of 5–240 min contact time, 10–100 mg 3K-SCB_750_ in 25 mL NOR solutions (corresponding to 0.4–4 g·L^−1^), pH 3–11, equilibrium concentrations range of 100 to 900 mg·L^−1^, and a temperature range of 10 to 40 °C. After shaking for a certain period of time, the suspensions were centrifuged at 3000 rpm for 5 min. The supernatants were then collected and analyzed for NOR with a UV−VIS-1100 spectrophotometer at 273 nm (Meipuda, Shanghai, China).

The extent of NOR removal was calculated using the equation R = [(C_0_ − C_e_)/C_0_] × 100% [18], where R is the percentage of NOR removal, C_0_ is the initial concentration, and C_e_ is the equilibrium concentration (both in mg·L^−1^). Blank experiments (without adsorbents) were also carried out for comparison. All of the experiments were performed in duplicate.

### 2.3. Characterization of Biochars

Based on the preliminary adsorption experiments, the best KOH-modified biochar (i.e., 3K-SCB_750_) was chosen for further instrumental characterization and comparison with the unmodified biochar. The surface morphology and microstructure of 3K-SCB_750_ and unmodified biochars were examined using a JEOL scanning electron microscope (FE-SEM, JSM-7800F, Tokyo, Japan). The specific surface area (SSA) and micro- and nanopores were determined using the N_2_ adsorption method (BET, Tristrar II 3020, Micrometric, Norcross, USA). The biochars were also subjected to X-ray powder diffraction analysis (XRD, Bruker D8, Mannheim, Germany) equipped with Cu K_α_ radiation and operated at 40 kV and 40 mA. All the XRD data were collected at a step size of 0.05° and counting time of 0.6 s per step. Transmission infrared spectra were obtained using a Fourier transform infrared spectrometer (FTIR, Nicolet 20, Thermo Scientific, Waltham, MA, USA). Each FTIR specimen was prepared by pressing a mixture of 1–2 mg of modified biochar and 200 mg of KBr into a small disc.

### 2.4. Theory of Sorption

#### 2.4.1. Sorption Isotherms

Both Langmuir (Equation (1)) and Freundlich (Equation (2)) isotherm models were used to fit the adsorption data. The Langmuir model assumes adsorption of a monolayer onto finite homogenous active sites, while the Freundlich model considers a multilayer adsorption process. The two isotherm models are expressed as follows [3,18,19]:

(1) $$\frac{C_{e}}{Q_{e}}=\frac{C_{e}}{Q_{0}}+\frac{1}{Q_{0}K_{L}}$$

(2) $$\ln\;Q_{e}=\ln\;K_{F}+\frac{1}{n}lnC_{e}$$

where Q_e_ (mg·g^−1^) is the adsorption capacity at equilibrium and is calculated by Q_e_ = (C_0_ − C_e_) × V/W (V is the solution volume in L and W is the mass of the adsorbent in mg), Q_0_ (mg·g^−1^) is the maximum adsorption capacity, C_e_ (mg·L^−1^) is the equilibrium concentration of NOR, K_L_ (L·mg^−1^) is a constant related to the sorption energy, K_F_ ((mg/g) · (L/mg)^1/n^) is the Freundlich constant associated with the relative capacity, and n relates to the sorption intensity.

The dimension parameter of the equilibrium or adsorption intensity (R_L_) of the Langmuir equation can be further re-written as follows [20]:

(3) $$R_{L}=\frac{1}{\left(1+K{\times C}_{0}\right)}$$

where C_0_ (mg·L^−1^) is the initial concentration of NOR, R_L_ is an indicator of sorption, and K (L·mg^−1^) is a constant related to the adsorption energy. The value of R_L_ indicates the type of isotherms: R_L_ > 1 for unfavorable sorption, R_L_ = 1 for linear sorption, 0 < R_L_ < 1 for favorable sorption, and R_L_ = 0 for irreversible sorption.

#### 2.4.2. Adsorption Kinetics

In this study, pseudo-first-order, pseudo-second-order, and intraparticle diffusion models were employed to analyze the kinetic data. The first-order kinetic model is given as follows [21]:

(4) $$\frac{1}{Q_{t}}=\frac{k_{1}}{Q_{e}t}+\frac{1}{Q_{e}}$$

The pseudo-second-order kinetic model describes the sorption mechanism and can be expressed by the following equation [20]:

(5) $$\frac{t}{Q_{t}}=\frac{1}{k_{2}Q_{e}^{2}}+\frac{t}{Q_{e}}$$

where Q_e_ and Q_t_ are the amounts of NOR adsorbed on the adsorbent at equilibrium (in mg·g^−1^) and at various times t, respectively, and k_1_ is the rate constant (min^−1^) of the first-order model for the sorption process. k_1_ is calculated from the slope of the 1/Q_t_ versus 1/t plot and k_2_ (g/(mg·min)) represents the rate constant of the sorption. k_2_ and Q_e_ can be calculated from the plot of t/Q_t_ against t.

The intraparticle diffusion model can be described by the equation below [22]:

(6) $$Q_{t}=k_{3}t^{\frac{1}{2}}+C$$

where k_3_ (mg·g^−1^·min^−1/2^) represents the rate constant of the intraparticle diffusion, t is time in minute, and C is the intercept.

Thermodynamic parameters such as standard free energy change (ΔG^0^), standard enthalpy change (ΔH^0^), and standard entropy change (ΔS^0^) can be calculated using the following equation [20,22]:

(7) $$\ln\;K_{c}=-\frac{\Delta G{}^{\circ}}{RT}=\frac{\Delta S{}^{\circ}}{R}-\frac{\Delta H{}^{\circ}}{RT}$$

where K_c_ = Q_e_/C_e_. ΔG^0^, ΔH^0^, and ΔS^0^ can be calculated from the plot of lnK_c_ versus 1/T.

## 3. Results and Discussion

### 3.1. Characterization

#### 3.1.1. SEM

**Figure 2:**
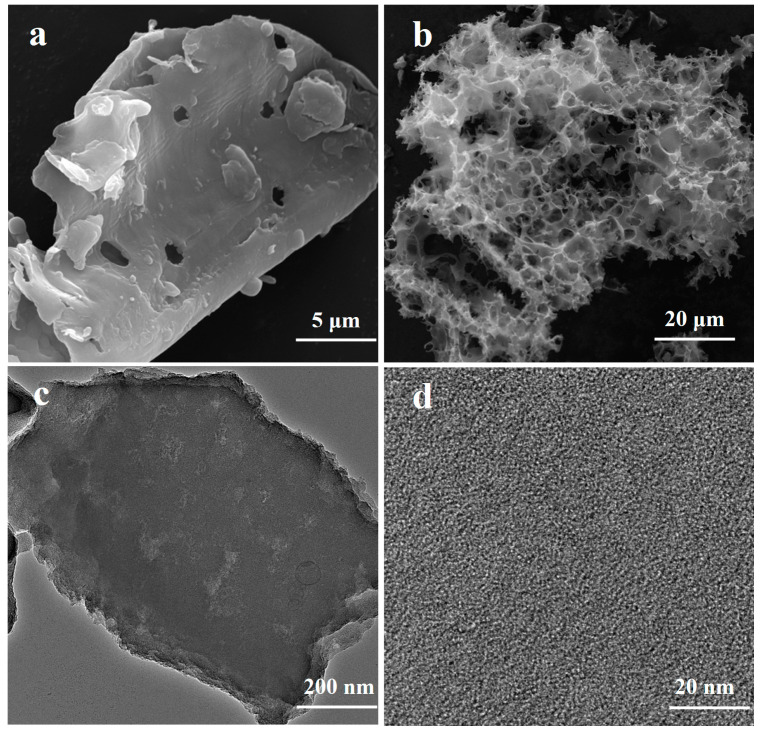
SEM images of the unmodified biochar B_750_ (**a**) and 3K-SCB_750_ (**b**), and TEM (**c**) and HRTEM images (**d**) of 3K-SCB_750_.

The SEM micrographs of the unmodified biochar (B_750_) and the KOH-modified 3K-SCB_750_ biochar are shown in Figure 2. A smooth and layered surface morphology with several small pores was found on B_750_, as it contained cellulose. Compared with B_750_ (Figure 2a), 3K-SCB_750_ showed a 3D hierarchical porous architecture with more random, open pores and cracks (Figure 2b). The KOH activation mechanism is mainly associated with an internal reaction that releases gas and forms pores. The specific surface area of B_750_ was measured to be 513 m^2^·g^−1^, much smaller than that of 3K-SCB_750_ (1039 m^2^·g^−1^), suggesting that KOH activation is an effective method for enhancing porous structures and increasing the specific surface area of the biochar material. This may be due to the activation reaction between KOH and the carbonaceous structure occurring above 700 °C, which is known to promote the development of the pore structure of porous carbon [23,24].

As shown in the TEM image (Figure 2c), the 3K-SCB_750_ sheet contains numerous pores of a nanoscale and in different sizes. A high-resolution TEM (HRTEM, Figure 2d) image further reveals that numerous nanopores exist in the carbon skeletons of 3K-SCB_750_. Such a unique hierarchical porous structure is beneficial to adsorption [25]. The formation of pores occurs in three stages [23]: (i) etching of carbon (precursor) by KOH, creating rudimentary porous network; (ii) development of pores by gasification of carbon, producing CO_2_ and CO; and (iii) intercalation of K from KOH in the carbon matrix and expansion of the carbon network. The intercalated K is then removed through continuous washing, thereby showing nanopores within the enlarged carbon network.

#### 3.1.2. BET

**Figure 3:**
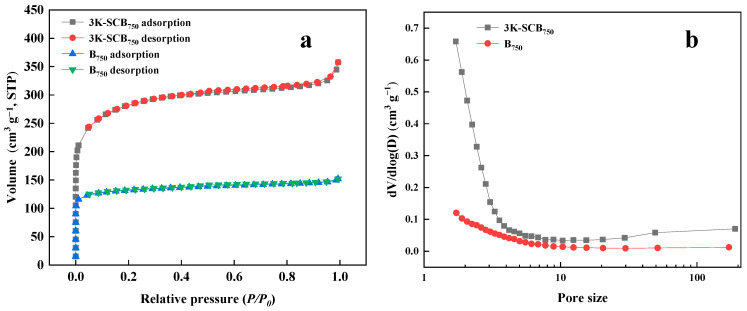
The porosity characteristics of B_750_ and 3K-SCB_750_: (**a**) Ar adsorption/desorption plots and (**b**) pore size distribution.

As the surface area of porous structures is the key to high-performance adsorbent materials, Ar adsorption/desorption tests of B_750_ and 3K-SCB_750_ were carried out to demonstrate the pore structure characteristics (Figure 3 and Table 1). As shown in Figure 3a, the isotherm profiles of B_750_ and 3K-SCB_750_ were all assigned as type II and type IV with a rapid adsorption of Ar at low relative pressures (P/P_0_ < 0.03), indicating the presence of a large amount of micropores [21]. From P/P_0_ = 0.03 onwards, the amount of adsorption increased slowly until P/P_0_ reached 0.3, suggesting the formation of small-sized mesopores [26]. The average pore size of B_750_ and 3K-SCB_750_ was calculated to be 1.76 and 1.94 nm, respectively, using the Brunauer–Emmett–Teller (BET) and Barrett–Joyner–Halenda (BJH) models (Figure 3b and Table 1). The BET surface area and pore volume of 3K-SCB_750_ were calculated to be 1039 m^2^·g^−1^ and 0.50 cm^3^·g^−1^, respectively, approximately twice that of B_750_. The large specific surface area with hierarchical pores would provide a vast number of accessible sites for the fast adsorption of NOR.

#### 3.1.3. XRD, Rama and FTIR

**Figure 4:**
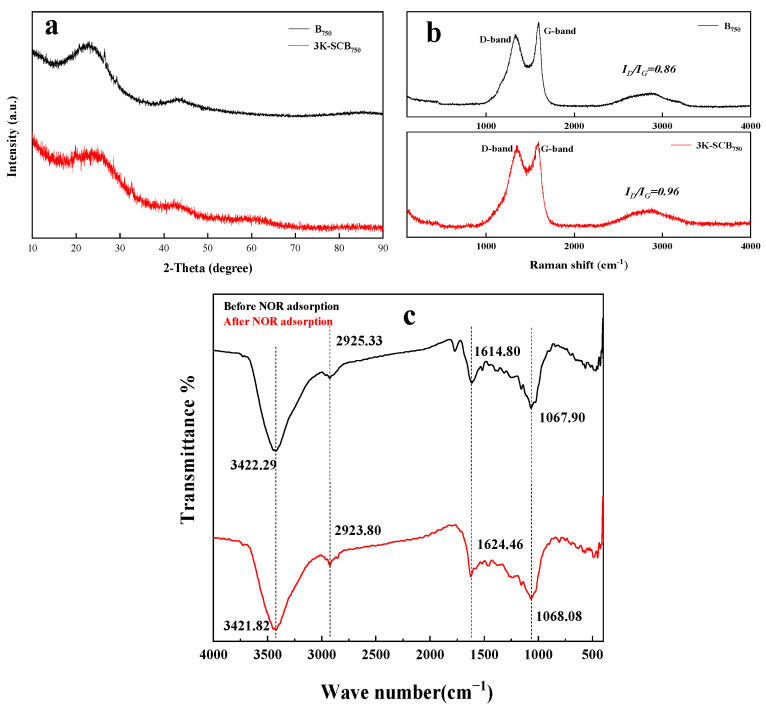
(**a**) The XRD patterns, and (**b**) Raman and (**c**) FTIR spectra for the unmodified and KOH-modified 3K-SCB_750_ biochars.

The XRD patterns of B_750_ and 3K-SCB_750_ in Figure 4a illustrate two broad peaks and weak peaks centered around 24.1° and 43.7°, respectively, both of which are considered characteristic peaks of graphitic carbon, clearly indicating that both materials are partly graphitized as well as having crystallinity to a certain degree [27,28]. Some small and sharp diffraction peaks from B_750_ due to the salting-out effect formed from water loss or cracking, while no more sharp peaks were observed for 3K-SCB_750_ [13]. Moreover, a decrease in the peak intensity at 24.1° in the 3K-SCB_750_ XRD diffraction pattern suggests that 3K-SCB_750_ was less graphitized than B_750_ [18].

The graphitic structure was also confirmed by Raman spectroscopy (Figure 4b). The intensity ratio of two peaks (I_G_/I_D_) were found to partially depend on the degree of graphitization [29]. Apparently, the Raman spectra of 3K-SCB_750_ and B_750_ revealed two typical peaks centered around 1335 and 1583 cm^−1^, which can be assigned to the D and G bands, respectively (Figure 4b). The characteristic D band can be attributed to the presence of disorder in the graphene structure, whereas the G band is due to the stretching band of the sp^2^-hybridized carbon. The relative intensity ratios of the D to G bands (I_D_/I_G_) for B_750_ and 3K-SCB_750_ were 0.86 and 0.96, respectively, suggesting the formation of less disordered graphite or defects in 3K-SCB_750_ due to corrosion by KOH [30,31]. Raman spectra also demonstrated a more defective structure and less graphitization of 3K-SCB_750_, consistent with the XRD analysis.

The FTIR spectra displayed the variations in functional groups on the surface of 3K-SCB_750_ before and after NOR adsorption (Figure 4c). The predominant peaks of clean 3K-SCB_750_ were found to be at 3422, 2925, 1615, and 1068 cm^−1^. The absorption peak at 3422 cm^−1^ was assigned to hydroxyl (-OH) groups, suggesting the presence of hydroxyl-containing structures (alcohol, phenol, and carboxylic acid) and moisture due to O-H stretching [8]. The three peaks at 2925 cm^−1^, 1615 cm^−1^, and 1068 cm^−1^ corresponded to C−H stretching vibrations, C=O stretching vibrations, and C-OH bending vibrations, respectively [32].

Two peaks around 3422 and 2923.8 cm^−1^ were shifted after the adsorption of NOR, possibly due to the deprotonation of carboxyl and hydroxyl groups, suggesting that hydrogen bonding may play an important role in the adsorption process [28]. Likewise, the band at 1615 cm^−1^ was shifted to 1624 cm^−1^, which originated from the interaction of π−π electron coupling between the NOR molecules and the aromatic rings of 3K-SCB_750_ [33]. In addition, these shifts implied that these oxygen-containing functional groups participated in the adsorption of NOR [34].

### 3.2. Adsorption Studies

#### 3.2.1. Adsorption of NOR Using Various Materials

**Figure 5:**
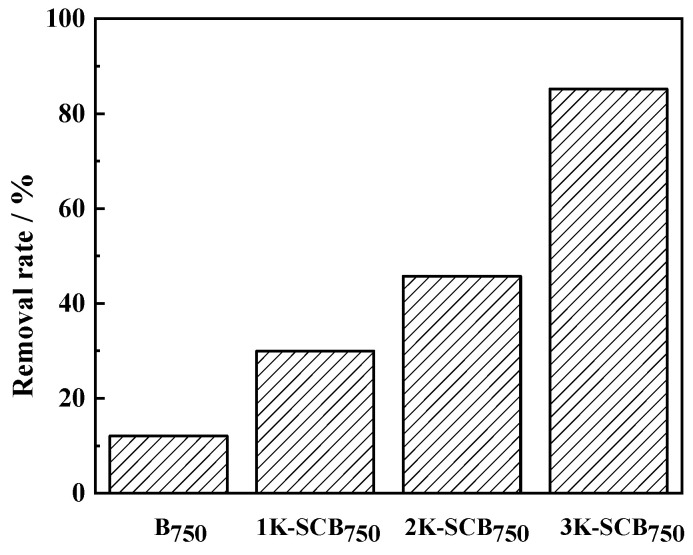
The percentage of NOR removal by different sorbents.

Here, 12%, 30%, 46%, and 85% of NOR were removed by B_750_, 1K-SCB_750_, 2K-SCB_750_, and 3K-SCB_750_, respectively (Figure 5). The results showed that 3K-SCB_750_ was the best material for the removal of NOR, while the unmodified material (B_750_) was the poorest-performing material. The good performance of the activated biochar was due to the formation of porous structures and large specific surface areas resulting from KOH activation. The effectiveness of the adsorption of NOR onto different sugarcane-bagasse-derived biochar was in the order of 3K-SCB_750_ > 2K-SCB_750_ > 1K-SCB_750_ > B_750_, corresponding to an increased adsorption performance with a decreasing solid-to-liquid mass ratio of bagasse-to-KOH.

#### 3.2.2. Effect of Contact Time

**Figure 6:**
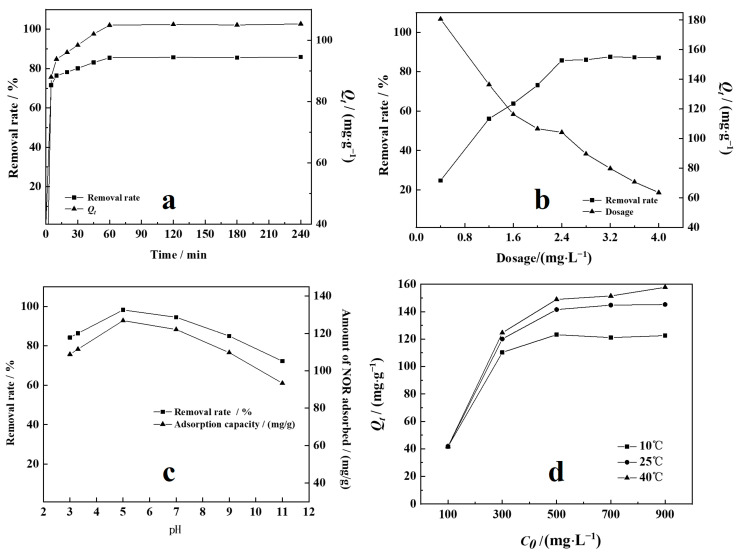
Effect of various experimental parameters on NOR removal by 3K-SCB_750_: (**a**) time, (**b**) 3K-SC B_750_ dosage, and (**c**) pH and (**d**) initial concentration of NOR and temperature. (**a**) Adsorbent dosage of 2.4 g·L^−1^, rotary speed of 300 rpm, initial pH 3.4, 25 °C, and initial NOR concentration of 300 mg·L^−1^. (**b**) Equilibration time of 60 min, initial pH 3.4, rotary speed of 300 rpm, 25 °C, and initial NOR concentration of 300 mg·L^−1^. (**c**) pH 3–11, adsorbent dosage of 2.4 g·L^−1^, contact time of 60 min, initial NOR concentration of 300 mg·L^−1^, and 25 °C. (**d**) Adsorbent dosage of 2.4 g·L^−1^, pH 5 (optimal pH based on results from (**c**)), equilibration time of 60 min, rotary speed of 300 rpm, and 25 °C.

Figure 6a illustrates the effect of contact time on the adsorption of NOR on 3K-SCB_750_. The initial adsorption of NOR was rapid and increased significantly within the first 15 min (Q_e_: 104.9 mg·g^−1^). No remarkable changes were observed after 60 min. Thus, the contact time of 60 min was used in the subsequent experiments. The initial rapid step (0–15 min) could be related to the rapid occupation of readily-accessible surface adsorption sites, whereas the subsequent slower step (15–60 min) may be ascribed to the formation of inner layer complexes [12,22].

#### 3.2.3. Effect of Adsorbent Dosage

The adsorption percentage and capacity (Q_e_) of NOR was found to be a function of 3K-SCB_750_ dosage under the conditions examined (Figure 6b). The NOR adsorption percentage increased from 24.8% to 87.1% and the adsorption capacity decreased from 180.7 to 63.5 mg·g^−1^ as the adsorbent dosage increased from 0.4 to 4 g·L^−1^. This was likely due to the increasing number of available adsorption sites with the increasing adsorbent dosage, resulting in a higher adsorption capacity, which was mainly attributed to the interactions between the particles (e.g., formation of aggregates leading to an increase in diffusion path length and a decrease in the total surface area of the adsorbent [20]). When the absorbent dosage increased from 2.4 to 4.0 g·L^−1^, the percentage of NOR adsorption only increased slightly. Hence, 2.4 g·L^−1^ adsorbent dosage was used in all of the subsequent experiments.

#### 3.2.4. Effect of pH

The effect of pH on the adsorption capacity of NOR by 3K-SCB_750_ was investigated at room temperature under the conditions of pH 3–11, adsorbate dosage of 2.4 g·L^−1^, and contact time of 60 min. Figure 6c shows that the adsorption of NOR onto 3K-SCB_750_ was highly pH-dependent. The amount of NOR adsorbed increased from 108.8 to 126.9 mg·g^−1^ as the pH increased from 3 to 5, but then decreased to 93.3 mg·g^−1^ from pH 5 to 11. This may be due to the different charge states of NOR under different pH conditions.

According to the physicochemical properties of NOR, it has two pK_a_ values (pK_a1_ = 6.34 and pK_a2_ = 8.75). At pH < pK_a1_, the main cationic form of NOR is NOR^+^ [12]. At pH ≥ pK_a1_, NOR exists in the form of zwitterionic NOR^±^ or neutral NOR^0^ [35]. When pK_a1_ < pH < pK_a2_, NOR is largely in the neutral NOR^0^ form and is adsorbed onto 3K-SCB_750_ via hydrogen bonding. At pH > pK_a2_, electrostatic attraction between 3K-SCB_750_ occurs when NOR is in the anionic form (NOR^−^). As ionization of NOR increases with increasing pH; this leads to a decrease in the adsorption of NOR on 3K-SCB_750_. The adsorption of NOR onto 3K-SCB_750_ may be dominated by electrostatic interactions. In addition, when the solution pH is acidic, the surface of the biochar is more easily protonated, thereby enhancing the strong hydrogen bonding between functional groups to promote the adsorption of NOR. At a low pH, NOR cations compete more intensely with protons for available active sites, hence decreasing the efficiency of NOR adsorption. The NOR adsorption decreased with increasing pH, which was partially caused by the competition between excess OH− in solution and anionic NOR. In brief, electrostatic force is one of the main mechanisms for NOR adsorption onto 3K-SCB_750_.

#### 3.2.5. Effect of the Initial Concentration and Temperature

Figure 6d highlights changes in the adsorption capacities at equilibrium (Q_e_) of NOR as a function of equilibrium concentrations in the range 100 to 900 mg·L^−1^ at three different temperatures. Initial increases in NOR concentration resulted in an increase in NOR adsorption by 3K-SCB_750_. The removal percentage of NOR increased rapidly when increasing the initial concentration from 100 to 500 mg·L^−1^, but only increased slightly and reached a stable condition when the initial concentration of NOR was between 500 and 900 mg·L^−1^. This is because the initial NOR concentration provided an important driving force to overcome all mass transfer limitations of NOR between the aqueous and solid phases. However, there was no distinct increase in Q_e_ values when the initial NOR concentration was greater than 500 mg·L^−1^, indicating that the adsorption reached saturation at a great NOR concentration due to a limited number of available surface binding sites [20]. According to Figure 6d, the increase in adsorption capacity and adsorption percentage with temperature was due to the increasing adsorption of NOR onto 3K-SCB_750_. This also indicates that the adsorption of NOR on 3K-SCB_750_ was endothermic.

### 3.3. Adsorption Kinetic Studies

#### 3.3.1. Adsorption Isotherms

The constants of three isotherms were obtained from the slope and intercept of the plots for each isotherm at different temperatures (Table 2). Among the isotherm models examined, the Langmuir isotherm was found to provide the best fit. The maximum adsorption capacity of NOR was estimated to be 157.4 mg·g^−1^, suggesting that 3K-SCB_750_ is a promising adsorbent for the removal of NOR from water. K_L_ increased with increased temperatures, indicating that the affinity of binding sites for NOR increased with temperature, and the values of R_L_ ranging from 0 to 1 suggest that the adsorption of NOR by 3K-SCB_750_ was kinetically favorable. In addition, R_L_ decreased from 0.024 to 0.002 as C_0_ increased from 100 to 900 mg·L^−1^. The magnitude of variation in RL (0 < R_L_ < 1) revealed that the adsorption of NOR by 3K-SCB_750_ was favorable and that 3K-SCB_750_ is a suitable adsorbent for removing NOR from aqueous solutions [23].

Furthermore, the comparison of our biochar material (3K-SCB_750_) with other previously reported biochars is shown in Table S1. The 3K-SCB_750_ derived from sugarcane bagasse with KOH modification exhibited an excellent NOR adsorption performance (157.4 mg·g^−1^) due to its large surface area and pore volume, as well as the abundant functional groups.

#### 3.3.2. Adsorption Kinetics

To investigate the adsorption rate and the adsorption mechanism, experiments were conducted as a function of time under the following conditions: dosage of 2.4 g·L^−1^, initial pH, equilibration time of 60 min, and rotary speed of 300 rpm. Pseudo-first-order, pseudo-second-order, and intraparticle diffusion models were used to fit the experimental results (Equations (4)–(6)). For surface adsorption reactions, the pseudo-first-order and pseudo-second-order models were evaluated to determine whether the solute interactions with the active sites on the 3K-SCB_750_ surface were physical or chemical. The parameters calculated from the kinetic models for NOR adsorption onto 3K-SCB_750_ are summarized in Table 3. The theoretical equilibrium adsorption capacity calculated using the pseudo-second-order kinetic model (123 mg·g^−1^) was close to the experimental equilibrium adsorption capacity, suggesting that the pseudo-second-order kinetic model is suitable for investigation of the adsorption behavior of NOR on 3K-SCB_750_. In addition, the pseudo-second-order kinetic model suggests that the adsorption mechanism may be chemically controlled rather than physically controlled, which was the main rate-limiting step throughout the adsorption process. The apparent adsorption rate constant (k) is an indicator for the adsorption rate, which is of great significance to rapid removal. Here, the k value (both k_1_ and k_2_) was less than 1, indicating the rapid NOR removal by 3K-SCB_750_.

**Figure 7:**
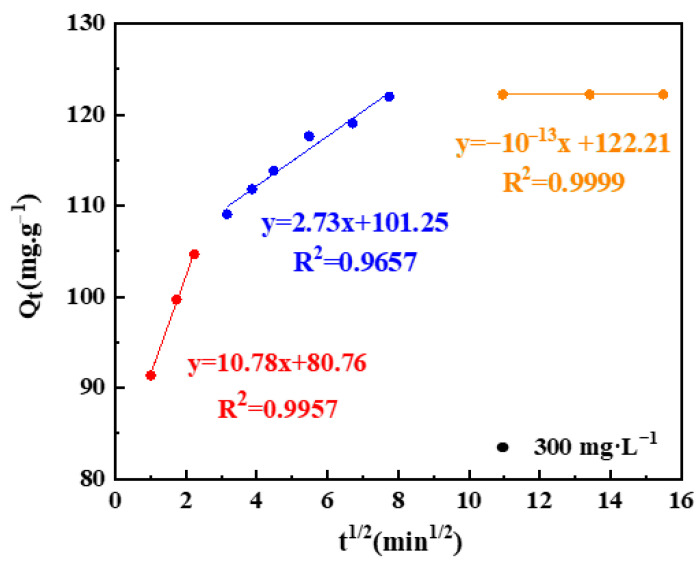
Intraparticle diffusion model for NOR adsorption onto 3K-SCB_750_ at different initial concentrations.

To identify the diffusion mechanisms influencing the adsorption of NOR particles onto 3K-SCB_750_, intraparticle diffusion model (IDM) was applied to examine the experimental data. A linear regression analysis of Q_t_ vs. t_1/2_ was also performed to obtain K_id_ and C constants. Figure 7 demonstrates that the adsorption process of NOR by 3K-SCB_750_ involved three steps (Table 4) throughout the entire course of the experiment, including the first external film diffusion stage, the second gradual adsorption stage, and the third equilibrium adsorption stage [36]. The R^2^ values obtained from these three steps were between 0.96 and 0.99, indicating that the IDM mechanism plays an important role in this adsorption process, but not the sole rate-limiting step (C_1_, C_2_, and C_3_ >0) [37]. However, data in Figure 7 were found to deviate from the origin, revealing that the NOR adsorption onto 3K-SCB_750_ involved intra-particle diffusion [6,10]. Moreover, the obtained diffusion rates in the order of k_id1_ > k_id2_ > k_id3_ implies that the external film diffusion is crucial during the whole diffusion process [38].

### 3.4. Thermodynamic Study

The thermodynamic parameters obtained at various temperatures from the Van’t Hoff plot and Gibbs free energy equation are given in Table 5. The energetics and spontaneity of the adsorption of NOR onto 3K-SCB_750_ were further explored using the standard free energy change (ΔG^0^), standard enthalpy change (ΔH^0^), and standard entropy change (ΔS^0^). The positive ΔH^0^ value (15.089 kJ·mol^−1^) suggests that the adsorption was an endothermic and physisorptive process, more favorable under elevated temperature conditions. The value and sign of ΔS^0^ also suggest whether the increasing (ΔS^0^ > 0) or decreasing randomness (ΔS^0^ < 0) at the solid−liquid phase occurred during adsorption. Generally, the absolute magnitude of change in Gibbs free energy for physical adsorption was smaller than that for chemisorption [22]. The ΔG^0^ values were calculated to be 1.24, 0.50, and −0.23 kJ·mol^−1^ at 10, 25, and 40 °C, respectively. It was observed that ΔG^0^ decreased with increasing temperature, suggesting that the increase in ΔG^0^ was favorable for the removal of NOR at greater temperatures.

### 3.5. Adsorption Mechanism

The adsorption mechanism of carbon materials on organic pollutants mainly involves a pore-filling effect, as well as π−π bond and electrostatic attractions [12,23,39,40,41]. The adsorption mechanisms responsible for the remarkable performance of 3K-SCB_750_ for NOR removal can be described as follows.

1. Compared with the unmodified biochar B_750_, KOH treatment promoted NOR adsorption by 3K-SCB_750_ with the largest specific surface area and large pore volume, implying that Q_e_ of NOR may be related to the surface area and pore volume of the KOH-modified biochar material. Thus, it is speculated that NOR uptake primarily occurred via the pore-filling mechanism.
2. In light of the above kinetic analysis, it is apparent that the adsorption of NOR by 3K-SCB_750_ was in alignment with both pseudo-second-order and intraparticle diffusion kinetic models, suggesting that both chemisorption and diffusion were the rate-controlling steps during the whole adsorption process. For the chemisorption mechanism, the adsorption of NOR occurred mainly by the electrostatic interactions between the positively and the negatively charged surfaces.
3. According to the XRD and Raman results, 3K-SCB_750_ was found to have a graphite structure, which could act as both electron donors and acceptors [42]. Furthermore, FTIR analysis revealed that hydrogen bonds and π−π electron coupling may also play an important role in the adsorption process.

### 3.6. Regeneration of Biochar

As far as the economic value and sustainability of the adsorbent are concerned, regeneration of used adsorbents is an essential consideration [28]. After the adsorption, it is vital to ensure efficient desorption in order for 3K-SCB_750_ to be a reusable material. After the initial NOR sorption, 3K-SCB_750_ was desorbed with anhydrous ethanol (3K-SCB_750_:ethanol = 1 g:100 mL). The removal efficiency of NOR by 3K-SCB_750_ was still over 65% after four adsorption–desorption cycles; the reduced removal efficiency was possibly due to irreversible clogging of the pores [43]. In general, the regeneration results suggest that 3K-SCB_750_ is a high-performance adsorbent with reliable reusability for practical applications in the future.

## 4. Conclusions and Recommendations

A novel biochar (3K-SCB_750_) was successfully fabricated via a convenient KOH-based activation pyrolysis at 750 °C using Chinese sugarcane bagasse and was used for the removal of norfloxacin from aqueous solutions. The as-prepared biochar with a large specific area of 1038.8 m^2^·g^−1^ exhibited an excellent NOR adsorption performance, resulting from its unique and well-developed microporous structure with an average pore size of 1.94 nm. The batch adsorption experiments showed that the adsorption of NOR onto 3K-SCB_750_ was pH-dependent. According to the best-fit Langmuir isotherm model, 3K-SCB_750_ had a significant NOR adsorption capacity of 157.4 mg·g^−1^ at 40 °C with monolayer adsorptions. The pseudo-second-order kinetic model and intraparticle diffusion kinetic models best described the adsorption behavior of NOR onto 3K-SCB_750_, suggesting that both chemisorption and diffusion were the rate-controlling steps over the whole adsorption process. The thermodynamic results suggest that the adsorption of NOR onto 3K-SCB_750_ was spontaneous and endothermic. Finally, the mechanism of the adsorption of NOR by 3K-SCB_750_ mainly involved the combination of the pore-filling effect and electrostatic attraction, as well as π−π bond coupling interactions. Overall, 3K-SCB_750_ was proven to be a promising adsorbent for practical applications in the treatment of water contaminated by antibiotics. Future work should assess the performance of this novel biochar for the remediation of other important organic micropollutants as multiple pollutant mixtures and in more complex solution matrices containing competing organic compounds.

## Figures and Tables

**Table 1:** Parameters of B_750_ and 3K-SCB_750_ from the BET analysis.

Sample	*S*_BET_ (m^2^·g^−1^)	V_total_ (cm^3^·g^−1^)	V_micro_ (cm^3^·g^−1^)	Average Pore Size (nm)
B_750_	513	0.23	0.17	1.76
3K-SCB_750_	1039	0.50	0.26	1.94

**Table 2:** The fitting parameters of the isothermal adsorption models.

Temperature (K)	Langmuir Model			Freundlich Model		
	Q_m_ (mg·g^−1^)	K_L_	R_L_^2^	1/n	K_F_	R_F_^2^
283	122.60	0.596	0.9999	0.132	58.029	0.927
298	145.71	0.804	0.9999	0.133	69.678	0.921
313	157.45	1.103	0.9992	0.119	81.484	0.701

**Table 3:** The fitting parameters of different kinetic adsorption models on NOR removal by 3K-SCB_750_.

C_0_ (mg·L^−1^)	Q_e_ (mg·g^−1^)	Pseudo-First-Order			Pseudo-Second-Order		
		Q_1_ (mg·g^−1^)	k_1_	R_1_^2^	Q_2_	k_2_	R_2_^2^
300	120.08	20.330	0.046	0.975	122.985	0.007	0.9999

**Table 4:** Intraparticle diffusion model parameters for NOR adsorption.

C_0_ (mg·L^−1^)	Step1 (1–5 min)			Step2 (10–60 min)			Step3 (120–240 min)		
	C_1_	K_id1_	R_1_^2^	C_2_	K_id2_	R_2_^2^	C_3_	K_id3_	R_3_^2^
300	80.74	10.78	0.9971	101.25	2.73	0.9657	122.21	10^−13^	0.9999

**Table 5:** Thermodynamic parameters for the adsorption of NOR by 3K-SCB_750_.

ΔH^0^ (kJ·mol^−1^)	ΔS^0^ (J·mol^−1^·K^−1^)	ΔG^0^ (kJ·mol^−1^)		
		10 °C	25 °C	40 °C
15.089	48.883	1.24	0.50	−0.23

## Data Availability

The data presented in this study are available on request from the first and corresponding author (Y.Z.). The data are not publicly available due to the privacy of research participants.

## Supplementary Materials

The following supporting information can be downloaded at: https://www.mdpi.com/article/10.3390/toxics11110908/s1, Table S1: Effect of various modified biochar for remediation of organic pollutants. References [44,45,46,47] are cited in the Supplementary Materials.
