# Supplementary material for: Effective Use of Sugarcane-Bagasse-Derived KOH-Activated Biochar for Remediating Norfloxacin-Contaminated Water
Source: Toxics. 2023 Nov 6;11(11):908. doi: 10.3390/toxics11110908 (PMC10674977; doi:10.3390/toxics11110908)
Supplement: Supplementary file 1 [file toxics-11-00908-s001.zip › toxics-2600764-supplementary.pdf]

Table S1. Comparison of various modified biochar materials for remediation of organic pollutants.

Biochar (Biomass)	Biochar modification	Biochar preparation condition	pollutant	Adsorption efficiency ($mg \cdot g^{-1}$)	References
GO-BC (Bamboo sawdust)		Pyrolysis at 600 °C	Sulfamethazine	23.42	S1 [1]
CRB (Crab shell)	Calcium-rich biomass	Pyrolysis at 800 °C	Chlortetracycline hydrochloride	70	S2 [2]
MRBC (Reed)	Magnetization	Pyrolysis at 600 °C	Florfenicol	9.29	S3 [3]
AMB (Corn stalks, reed stalks and willow branches)	$ZnCl_2$ and $FeCl_3$	Pyrolysis at 500 °C	Norfloxacin	7.6249	S4 [4]
APB (Potato stem and natural attapulgite)	—	Pyrolysis at 500 °C	Norfloxacin	5.24	S5 [5]
GAB (Algal)	H_3PO_4	Pyrolysis at 450 °C	Norfloxacin	86	S6 [6]
SCGB (Coffee grounds)	—	Pyrolysis at 500 °C	Norfloxacin	69.8	S7 [7]
**3K-SCB$_{750}$ (Sugarcane bagasse)**	**NaOH**	**Pyrolysis at 750 °C**	**Norfloxacin**	**157.4**	**This study**

Reference

1. Huang, D., et al., Sorptive removal of ionizable antibiotic sulfamethazine from aqueous solution by graphene oxide-coated biochar nanocomposites: influencing factors and mechanism. Chemosphere, 2017. 186: p. 414-421.
2. Xu, Q., et al., Interaction between chlortetracycline and calcium-rich biochar: Enhanced removal by adsorption coupled with flocculation. Chemical Engineering Journal, 2020. 382: p. 122705.
3. Zhao, H. and Y. Lang, Adsorption behaviors and mechanisms of florfenicol by magnetic functionalized biochar and reed biochar. Journal of the Taiwan Institute of Chemical Engineers, 2018. 88: p. 152-160.
4. Wang, B., et al., Preparation of biochar by simultaneous carbonization, magnetization and activation for norfloxacin removal in water. Bioresource Technology, 2017. 233.
5. Li, Y., et al., Removal of Norfloxacin from aqueous solution by clay-biochar composite prepared from potato stem and natural attapulgite. Colloids and Surfaces A: Physicochemical and Engineering Aspects, 2017. 514: p. 126-136.
6. Zhang, T., et al., Engineering mesoporous algal-based biochars for efficient remediation of norfloxacin pollution in marine environment. Environmental Advances, 2022. 9: p. 100302.
7. Nguyen, V.-T., et al., Adsorption of norfloxacin from aqueous solution on biochar derived from spent coffee ground: Master variables and response surface method optimized adsorption process. Chemosphere, 2022. 288: p. 132577.
